# Viscoat Assisted Inverted Internal Limiting Membrane Flap Technique for Large Macular Holes Associated with High Myopia

**DOI:** 10.1155/2016/8283062

**Published:** 2016-03-07

**Authors:** Zongming Song, Mei Li, Junjie Liu, Xuting Hu, Zhixiang Hu, Ding Chen

**Affiliations:** Eye Hospital, Wenzhou Medical University, 270 Xueyuan Road, Wenzhou, Zhejiang 325027, China

## Abstract

*Purpose*. To investigate the surgical outcomes of Viscoat® assisted inverted internal limiting membrane (ILM) flap technique for large macular holes (MHs) associated with high myopia.* Design*. Prospective, interventional case series.* Methods*. Fifteen eyes of 15 patients with high myopia underwent vitrectomy and Viscoat assisted inverted ILM flap technique to treat MH without RD. Patients were followed up over 6 months. The main outcome measures were MH closure evaluated by optical coherence tomography (OCT) and best-corrected visual acuities (BCVAs).* Result*. MH closure was observed in all eyes (100%) following the initial surgery. Type 1 closure was observed in 13 eyes (86.7%); type 2 closure was observed in the remaining 2 eyes (13.3%). Compared to the preoperative baseline, the mean BCVA (logarithm of the minimum angle of resolution) improved significantly at 3 months and 6 months after surgery (*P* = 0.025, 0.019, resp.). The final BCVA improved in 10 eyes (66.7%), remained unchanged in 3 eyes (20.0%), and worsened in 2 eyes (13.3%).* Conclusion*. Vitrectomy combined with Viscoat assisted inverted ILM flap technique is an effective treatment for large MHs in highly myopic eyes. It may increase the success rate of the initial surgery and enhance the anatomical and functional outcomes.

## 1. Introduction 

High myopia (spherical equivalent of refractive error of at least −6 diopters (D) and axial length of at least 26.5 mm) has high prevalence in China and accounts for 4.1% of the population [1, 2]. Highly myopic eyes are susceptible to different retinopathies, among which macular holes (MHs) with or without associated retinal detachment (RD) are one of the most vision-threatening complications [2]. The mechanism of MH is different in highly myopic eyes versus nonmyopic ones. Various factors such as severe axial elongation of the globe, the presence of posterior staphyloma, and chorioretinal atrophy make these cases challenging [3].

Vitrectomy has been the gold standard of MH treatment [4], and adjunctive procedures have been developed to improve the MH closure rate [3, 5, 6]. Recently, the inverted internal limiting membrane (ILM) flap technique was reported by Michalewska et al. [7] to have higher closure rate for MH, in which ILM around the MH was left to cover the MH to facilitate hole closure and regeneration of retinal structure. This technique was also applied to myopic MHs and proved to be beneficial in both the anatomical and functional outcomes [8, 9]. However, there are some limitations of the original technique. First, spontaneous retroversion of the ILM flap occurred frequently in up to 14%–20% of cases during the fluid-air exchange, leading to an initial failure [7, 9]. Repeated surgeries are implemented to reposition the flap to cover the MH, which increase the patient's pain and burden. Second, a rolled segment of the peeled ILM other than a single-layered ILM is used to fill the MH, which is less regular and physiological compared to the normal foveal contour and may induce over gliosis. Third, the indocyanine green (ICG) stained ILM is placed into the MH, which has macular toxicity and may induce retinal pigment epithelium (RPE) damage [10].

To overcome the drawbacks of the original inverted ILM flap technique as indicated above and to increase the success rate of the initial surgery for large MHs associated with high myopia, the authors developed a modified technique, that is, the vitrectomy combined with Viscoat assisted single-layered inverted ILM flap technique. The surgical outcomes of this modified technique for large MH without RD were evaluated in a highly myopic population.

## 2. Methods

This study was a prospective and interventional case series. Patients with large MHs associated with high myopia were enrolled in the Eye Hospital of Wenzhou Medical University from February 2014 to January 2015. The inclusion criteria were as follows: high myopia > −6 D; axial length > 26.5 mm; clinical presentation of MH without RD; minimum diameter of MH ≥ 400 *μ*m; intraocular pressure < 21 mmHg. Patients with a history of RD or proliferative vitreoretinopathy, any kind of retinal surgery, diabetic retinopathy, vitreous hemorrhage, retinal vascular occlusion, uveitis, trauma, optic atrophy, ocular tumors, glaucoma, corneal opacity, or incomplete chart records were excluded.

Each patient was informed about the risks and benefits of the study and their written informed consent was obtained. The study was approved by the Institutional Review Boards of Wenzhou Medical University. All investigations adhered to the tenets of the Declaration of Helsinki.

### 2.1. Surgical Technique

All eyes included in this study were treated with standard 23-gauge 3-port pars plana vitrectomy (PPV) by one experienced surgeon (Zongming Song) under local anesthesia. For this procedure, a posterior vitreous detachment was first created, followed by the removal of the residual thin premacular posterior cortex. The peripheral vitreous was also excised. Triamcinolone acetonide was used intraoperatively to facilitate visualization of the vitreous and posterior hyaloid in all eyes. Epiretinal membrane was removed with forceps if present.

**Figure 1:**
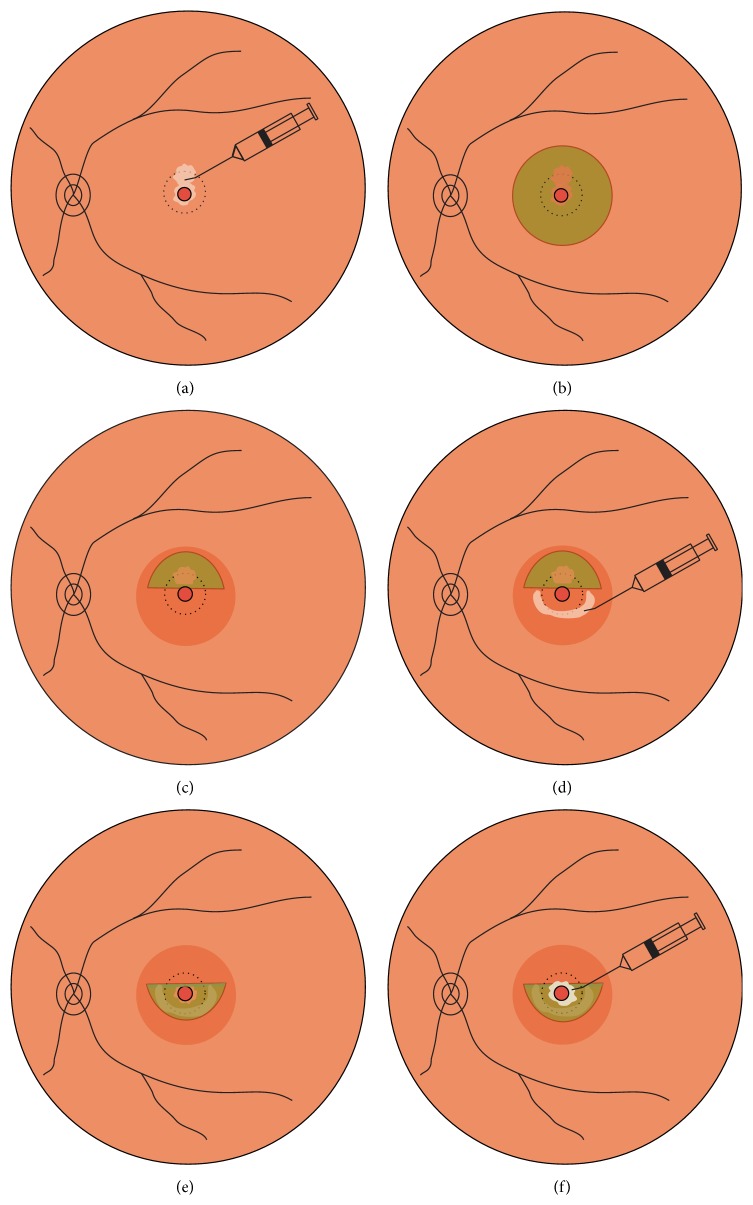
Schematic drawings showing the Viscoat assisted single-layered inverted internal limiting membrane (ILM) flap technique. (a) After performing a 23-gauge 3-port pars plana vitrectomy, Viscoat is injected into the macular hole (MH) and its mirror symmetrical area superior to MH. (b) Indocyanine green (ICG, 0.125% solution) is then applied around the MH within the arcade to stain the ILM. Excessive ICG as well as Viscoat is removed by suction. (c) The ILM is peeled off in a circular fashion for approximately 2.5 disc diameters around the MH. The inferior ILM is completely peeled off, while the superior ILM is not removed completely but left attached to the edge of the MH, forming an ILM flap of about 1 disk diameter. (d) Viscoat is injected and smeared in an arch around the MH in the lower half part of the macular as an adhesive. (e) The ILM flap is flipped by inverting it using the intraocular forceps to cover the whole MH and gently massaged to make it flattened. (f) Supplementary Viscoat is applied on top of the inverted ILM flap as ballast before air-fluid exchange is performed.

The inverted ILM flap technique was modified based on the method reported previously [7] with major improvement as described below. Viscoat (Alcon Laboratories, Fort Worth, TX, USA) was used during the procedure. It is a low-molecular-weight dispersive viscoelastic material composed of 3% sodium hyaluronate and 4% chondroitin sulfate, which has been commonly used in phacoemulsification to protect the corneal endothelial cells from the ultrasound damage. The initial use of Viscoat was prior to staining the ILM with ICG. Viscoat was injected into the MH and its mirror symmetrical area superior to the MH (Figure 1(a)). 0.125% ICG solution was then carefully applied around the MH within the arcade (Figure 1(b)). Excessive ICG as well as Viscoat was then removed by suction. Thereafter, the ILM was peeled off in a circular fashion for approximately 2.5 disc diameters around the MH (Figure 1(c)). During the half-circumferential peeling, the inferior ILM was completely peeled off while the superior ILM was not removed completely from the retina but left attached to the MH margin, forming an ILM flap of about 1 disk diameter. Viscoat was then injected and smeared in an arch around the MH in the lower half part of the macular as an adhesive (Figure 1(d)). Instead of packing the MH with a folded ILM reported by other authors [7, 11], the ILM flap was flipped by inverting it using the intraocular forceps to cover the whole MH and gently massaged to make it flattened (Figure 1(e)). Supplementary Viscoat was applied on top of the inverted ILM flap as ballast (Figure 1(f)). Air-fluid exchange was performed with gas tamponade by 15% perfluoropropane (C3F8) at the end of surgery. (See Supplementary video file S1 in Supplementary Material available online at http://dx.doi.org/10.1155/2016/8283062.) The patients were subsequently kept in a facedown position overnight for 3 days and were allowed to position themselves in any manner other than the supine position until the gas was absorbed.

### 2.2. Ophthalmic Examinations

All patients underwent a complete ophthalmic examination including refraction and best-corrected visual acuity (BCVA) measurement, slit-lamp examination, indirect ophthalmoscopy, and spectral domain optical coherence tomography (OCT) (SPECTRALIS HRA OCT; Heidelberg Engineering, Heidelberg, Germany) before surgery and at 1 week and 1, 3, and 6 months after surgery. B-Scan ultrasonography (B-Scan-Cinescan, Quantel Medical, France) and IOLMaster (Carl Zeiss Meditec AG, Jena, Germany) measurements were also performed before surgery. The BCVA was recorded in decimal acuity and converted to the logarithm of the minimal angle of resolution (log MAR) for statistical analysis. If the BCVA was counting fingers or hand movements, it was assigned as the equivalent Snellen acuity of 20/2000 or 20/20000, respectively, based on a previous publication [12].

### 2.3. Statistics

Statistical analysis was performed by SPSS for Windows version 17.0 (SPSS, Inc., Chicago, IL). The preoperative and postoperative BCVAs (logMAR values) were analyzed using the paired *t*-test. A *P* value of < 0.05 was considered to be statistically significant.

## 3. Results

The baseline clinical characteristics of all patients in this study are shown in Table 1. Six males and 9 females with a mean age of 60.1 ± 8.8 years were enrolled. The mean axial length was 29.83 ± 1.96 mm. All eyes had staphyloma, among which, 10 eyes had posterior pole staphyloma (type 1) and 5 eyes had macular staphyloma (type 2), as per the classification of Curtin [13]. The mean minimal diameter of the MH was 597.60 ± 115.84 *μ*m. Six eyes had retinoschisis around MH as shown in OCT examination. Eleven eyes were phakic, 1 eye was aphakic, and 3 eyes were pseudophakic before the surgery. Phacoemulsification with intraocular lens implantation was performed in 8 eyes concurrent with PPV and in 2 eyes during the follow-up period because of the development of cataracts. The mean follow-up period was 8.7 ± 2.0 months (range: 6.0–11.2 months).

**Figure 2:**
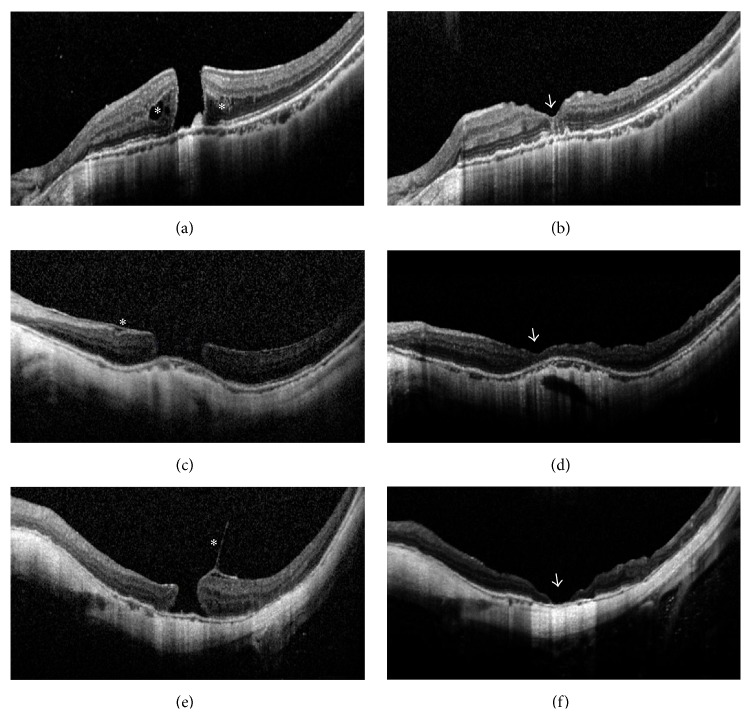
Optical coherence tomography (OCT) images of large myopic macular holes (MHs) in selected cases before and after surgery. (a) Case Number 5 before surgery. An MH of 563 *μ*m and retinoschisis around the MH (asterisks) was present. Best-corrected visual acuities (BCVAs) = 0.02. (b) Case Number 5 at 6 months after surgery. Type 1 closure in U-shape and restoration of the inner and outer segment junction line was achieved (arrowhead). BCVA = 0.3. (c) Case Number 12 before surgery. A large-sized MH of 812 *μ*m and epiretinal membrane (asterisk) was present. BCVA = FC/50 cm. (FC = finger counting) (d) Case Number 12 at 6 months after surgery. Type 1 closure in shallow V-shape was present (arrowhead). BCVA = 0.3. (e) Case Number 14 before surgery. A longstanding MH of 491 *μ*m and vitreomacular traction (asterisk) was present. BCVA = HM/BE. (HM = hand motion; BE = before eye) (f) Case Number 14 at 6 months after surgery. Type 2 closure with bare retinal pigment epithelium in the center (arrowhead) was present. BCVA = HM/BE.

There was no significant intraoperative complication that occurred in any case. No retroversion of the inverted ILM flap during fluid-air exchange was observed in any surgery. The main surgical results are listed in Table 2. MH closure was achieved in all 15 eyes (100%) after the initial PPV with Viscoat assisted inverted ILM flap technique. According to MH closure type classification based on OCT observation suggested by Kang et al. [14], type 1 closure (closed without foveal neurosensory retinal defect) was observed in 13 eyes (86.7%), among which 9 eyes had U-shape closure (Figures 2(a) and 2(b)) and 4 eyes had V-shape closure (Figures 2(c) and 2(d)); type 2 closure (closed with foveal neurosensory retinal defect) was observed in the remaining 2 eyes (13.3%) (Figures 2(e) and 2(f)). No development of the preexisting chorioretinal atrophy or any sign of new RPE damage was observed in any case.

The mean preoperative BCVA was 1.28 ± 0.70. The mean BCVA at 1 week and 1, 3, and 6 months after surgery was 1.31 ± 0.96, 1.19 ± 0.84, 1.10 ± 0.75, and 1.07 ± 0.88, respectively. In general, compared to the preoperative baseline, there was a significant improvement in BCVA at 3 months (*P* = 0.025) and 6 months (*P* = 0.019) after surgery, but not at 1 week and 1 month after surgery. The postoperative BCVA improved in 10 eyes (66.7%), remained unchanged in 3 eyes (20.0%), and worsened in 2 eyes (13.3%) at the final follow-up examination.

## 4. Discussion

Michalewska et al. [7] first introduced the inverted ILM flap technique for idiopathic MHs and proved its efficacy in improving MH closure rate and postoperative BCVA. They hypothesized that this inverted ILM could serve as a scaffold for the proliferation of glial cells that fill MH, producing an environment for the photo receptors to assume new positions in direct proximity to the fovea [7, 14]. This technique was then applied to more complicated cases such as chronic refractory MHs and myopic MHs, with or without RD [8, 11, 15–18]. Most of these reported outcomes were promising in both anatomical and functional aspects. However, there are still some challenges in this technique.

The most challenging part of this technique is maintaining the inverted ILM flap in position during the subsequent manipulation of fluid-air exchange. The free end of the ILM flap tends to turn over and move away from the hole opening, leading to a failure of the initial surgery. Michalewska et al. [7] reported spontaneous retroversion of the ILM flap during the fluid-air exchange in up to 14% of their cases. Kuriyama et al. [9] used inverted ILM flap technique for MHs associated with high myopia and failed in 20% of cases due to the complete detachment of flap. Repeated surgeries are required to reposition the flaps with the tamponade of silicone oil, which may aggravate the patient's pain and burden. Though the failure may be attributable to the surgeon's skill and learning curve, improvement to the original technique is required to increase the success rate of the initial surgery. Recently, Shin et al. [19] applied perfluorocarbon liquid on top of an inverted ILM flap before fluid-air exchange to ensure the proper covering of MH. This technique seems technically difficult and time-consuming. The fluid-air exchange needs to be performed very slowly with extreme care since minor turbulence may displace the liquid bubble and thereby dislocate the ILM flap. In addition, it takes extra time for the liquid to evaporate completely at the end of the fluid-air exchange. More recently, Lai et al. [11] proposed an idea of using autologous blood clot to stabilize and seal the ILM flap within MH before air-fluid exchange and claimed a success rate of 96% in their study. However, there may be potential risks of an increased rate of infection or proliferative vitreoretinopathy due to the extra manipulations and the introduction of blood from outside the eye. In our technique, a small amount of Viscoat was applied around MH and on top of the inverted ILM flap, which has dual effect of adhesive and ballast to stabilize the flap during the fluid-air exchange. Viscoat can be left in place without causing any toxic effect to the retina [20]. No dislodged inverted ILM flap was observed during or after the initial surgery in our cases.

Another difference from the original inverted ILM flap technique is that a large single-layered inverted ILM flap was used to cover the MH in our study. In the original technique, a rolled segment of the peeled ILM over the MH is massaged from all sides until the ILM is inverted and then packed into the MH [7]. Actually, the MH is filled with a roll of multilayered ILM rather than a single layer of inverted ILM flap, which has been confirmed in the postoperative optical coherence tomography [7, 15]. More recently, some authors intentionally repositioned or inserted a sufficient amount of inverted ILM tissue into the MHs to enhance the anatomical closure [11, 17]. A pack of multilayered ILM may be beneficial in anatomical recovery by sealing and bridging the hole. However, the appropriate amount of the folded ILM is difficult to determine, and too much ILM inserted in the center of macula may induce over fibrosis and hinder further functional recovery [19]. In addition, maneuvering the ILM within MH is technically difficult and may damage the foveal tissue. In our method, we half-circumferentially peeled the superior ILM to form an ILM flap of about 1 disk diameter size and covered the MH from its superior margin considering gravity. Therefore, the MHs were covered with single-layered inverted ILM flaps that were much larger than those in the original technique. We believe that the single-layered ILM over MH could provide a more regular and physiological structure for glial proliferation and aid in MH closure without inducing too much fibrosis in the fovea.

An important concern about the inverted ILM flap technique is the potential macular toxicity from the staining dye. Due to the limited availability of Brilliant Blue-G (BBG), ICG remains the most popular dye to facilitate ILM peeling used by Chinese retina specialists. The toxicity of ICG and its damage to retina after vitreoretinal surgery has been reported [21]. In case of subretinal application, RPE can be affected. Imai and Azumi [10] observed an expansion of RPE atrophy at 1 week after PPV with inverted ILM flap technique. They assumed that the staining of ILM with ICG and placing the ICG-stained tissue into MH may induce chorioretinal toxicity, provoking the RPE damage. To minimize the potential toxicity of ICG, the most effective way is to insulate the exposed RPE from ICG staining. In our technique, Viscoat was injected into the MH and the proposed covering portion of the inverted ILM (mirror symmetrical area superior to MH) before the injection of ICG solution. There was no development of the preexisting chorioretinal atrophy or any sign of new RPE damage observed in any case during the follow-up period.

The anatomical closure rate of myopic MHs after the initial surgery was 100% in our case series, which is significantly higher than those reported previously [8, 9, 11, 17]. In highly myopic eyes, the presence of staphyloma results in the shortening of retina in comparison to the posterior eye wall, which makes the surgical repair of MHs more challenging. As in the classic macular surgery, ILM peeling alone may release the tangential traction, but it does not compensate for retinal shortening in high myopia. The effective approach to overcome the discrepancy between the retina and sclera may be covering the MH with a large single-layered ILM flap as shown in our technique. There are multiple factors influencing the success rate of MH surgery, including the characteristics of each specific case, the surgical technique, and equipment adopted, as well as surgeon's skill and learning curve. We believe that the modification to the inverted ILM flap technique may contribute to the high success rate in the current study.

With regard to the visual outcome, the postoperative BCVA varied among the individuals despite the high anatomical success rate achieved in our study. Qu et al. [22] suggested that the visual outcomes might not be certain to improve in highly myopic MHs even if the MHs were anatomically closed. The MH closure type may be correlated to the final visual outcome [14, 23]. 86.7% of our cases had type 1 closure with a close to normal foveal configuration in U-shape or V-shape after surgery, which in general had significantly improved vision. Two cases presented as the type 2 closure with bare RPE in the center had unsatisfied visual outcome. The photoreceptor and external limiting membrane was absent based on the observation with OCT at the end of follow-up. Other factors accounting for the limited vision improvement may include the large size of MH, ultralong axial length, preexisted severe chorioretinal atrophy, presence of retinoschisis around MH, and complicated cataract. Further study is warranted to determine the risk factors that hinder the postoperative visual recovery in myopic MHs.

This study has some limitations, which include the small sample size, lack of a control group, and a relatively short follow-up period. In addition, this technique was only applied to the myopic MHs with attached retina in the current study. Further randomized controlled clinical studies involving a larger number of patients are needed to determine the efficacy of this technique in the management of myopic MHs with or without retinal detachment.

In conclusion, vitrectomy combined with Viscoat assisted inverted ILM flap technique is an effective treatment for large MHs without RD in highly myopic eyes. The proper use of Viscoat can effectively prevent retroversion of the ILM flap during the fluid-air exchange and minimize the toxic effect of ICG staining to the RPE. This technique may increase the success rate of the initial surgery and enhance the anatomical and functional outcomes. We highly recommend this simple, cost-effective technique for large MHs associated with high myopia.

## Supplementary Material

Video clips demostating the basic procedure of the Viscoat assisted single-layered inverted internal limiting membrane (ILM) flap technique. The vitreous is excised with the assistance of Triamcinolone acetonide. Viscoat^®^ (Alcon Laboratories, Fort Worth, Texas, USA) is injected into the MH before applying the 0.125% Indocyanine green (ICG) solution to the posterior pole. Excessive ICG and Viscoat is removed by suction.The ILM is peeled off in a circular fashion for approximately 2.5 disc diameters around the MH. The inferior ILM is completely peeled off while the superior ILM flap is inverted using the intraocular forceps to cover the whole MH. Supplementary Viscoat is applied on top of the inverted ILM flap as ballast before air-fluid exchange is performed.

## Figures and Tables

**Table 1:** Baseline clinical characteristics of highly myopic patients with large macular holes (*n* = 15).

Case number	Age (y)	Sex	Eye	Preop BCVA (Snellen)	Axial length (mm)	Subtype of staphyloma	MH diameter (*µ*m)	Retinoschisis around MH	Preop lens status
1	48	Female	os	0.2	30.08	Type 1	495	−	Phakia
2	54	Male	os	FC/50 cm	31.27	Type 1	471	−	Aphakia
3	49	Male	os	0.05	29.98	Type 1	521	−	Phakia
4	63	Female	os	0.04	31.94	Type 2	832	+	Phakia
5	67	Male	od	0.02	27.32	Type 2	563	+	Phakia
6	49	Female	od	0.3	27.64	Type 1	487	−	Phakia
7	78	Male	od	0.2	28.15	Type 2	633	+	Phakia
8	63	Female	os	0.16	28.27	Type 1	574	−	Pseudophakia
9	58	Female	od	0.05	30.66	Type 1	659	+	Phakia
10	55	Female	os	0.02	32.17	Type 2	708	+	Phakia
11	60	Male	os	0.1	33.16	Type 1	578	+	Pseudophakia
12	61	Female	od	FC/50 cm	28.42	Type 1	812	−	Pseudophakia
13	58	Male	od	0.3	27.65	Type 1	499	−	Phakia
14	63	Female	os	HM/BE	32.10	Type 2	491	−	Phakia
15	75	Female	os	0.05	28.62	Type 1	641	−	Phakia

MH = macular hole; BCVA = best-corrected visual acuity; Preop = preoperative; FC = finger counting; HM = hand motion; BE = before eye.

**Table 2:** Surgical results among all cases (*n* = 15).

Factor	
MH closed (number)	15
MH close type at last visit, number (%)	
Type 1	13 (86.7%)
Type 2	2 (13.3%)
Postoperative BCVA (logMAR)	
1 week	1.31 ± 0.96
1 month	1.19 ± 0.84
3 months	1.10 ± 0.75^*∗*^
6 months	1.07 ± 0.88^*∗*^
BCVA at last visit, number (%)	
Improved	10 (66.7%)
No change	3 (20.0%)
Worse	2 (13.3%)
Cataract surgery (number)	
Concurrent with PPV	8
During follow-up	2

MH = macular hole; BCVA = best-corrected visual acuity; logMAR = logarithm of the minimum angle of resolution; PPV = pars plana vitrectomy. ^*∗*^ *P* < 0.05.
